# Comments on “Until when will we grant specialist titles to doctors without medical residency?”

**DOI:** 10.1590/0100-6991e-20243844-en

**Published:** 2024-11-26

**Authors:** RODRIGO CARIRI CHALEGRE DE ALMEIDA, FERNANDO ANTONIO MENEZES DA SILVA

**Affiliations:** 1 - Universidade Federal de Pernambuco, Centro de Ciências Médicas - Recife - PE - Brasil; 2 - Comissão Nacional de Residência Médica - Brasília - DF - Brasil

Specialists and specializations: the need for a new regulatory framework in Brazil

The initiative of the Brazilian College of Surgeons to open space for the debate on the training of specialists in Brazil is not only timely, but essential. In the editorial published in volume 51, Gerson Alves Pereira Jr. presents compelling arguments in favor of qualifying the tests as specialists’ titles and reasons for physicians to undergo rigorous exams^1^. In the section of letters to the editor, Francisco Arsego questions the current model and suggests that the test be applied exclusively to graduates of medical residency programs, arguing that this training should be the only path to medical specialization in Brazil^2^.

The arguments presented in the articles reflect legitimate concerns about the medical education system in Brazil. Medical residency, globally recognized as the gold standard for the training of specialists, is a model that provides practical training under supervision in certified environments. This framework is key to ensuring that practitioners acquire not only theoretical knowledge but also essential skills such as crisis management, communication, collaboration, and reflection on clinical practice.

Initially, it is important to highlight two factors that contributed to the origin of our current model. Higher education and research in Brazil began late, which generated a need for recognition of the merit of the pioneers. Due to the characteristics of Portuguese colonization, Brazil was the last country in the Americas to offer higher education. Three hundred and eight years separate the arrival of the first Portuguese delegation from the creation of the first higher education courses. For more than one hundred and twenty years, these courses were offered only by isolated colleges, located in the great colonial centers, such as Salvador, Recife, and Rio de Janeiro. 

The young Brazilian universities emerged from the merger and federalization of the old colleges. This phenomenon may be intrinsically related to the successive ruptures of the democratic order and to the periods in which the country was under authoritarian regimes (Old Republic - 1889-1930, Vargas Dictatorship - 1930-1945, Military Dictatorship - 1964-1985)^3^.

Until 1808, the year in which the first two medical courses were created in Brazil (in Salvador and Rio de Janeiro), all doctors working in the country were trained abroad. The third course originated only 90 years later, in Porto Alegre. In this period, the Brazilian population increased from 4 million inhabitants in 1808 to 14 million in 18983. The current regulatory framework for professional practice in Brazil was constituted in a context of exception. Law No. 4,769, which creates professional councils, was enacted in 1965, and Law No. 6,932, which institutes the regime for the training of specialists through medical residency, dates from 1981.

Although the first residency programs date back to the mid-1940s, it was only in 1977 that the National Commission for Medical Residency was created, which defined residency as a way of training specialists through in-service training. For 169 years, all physicians trained in Brazil were generalists and became specialists by practice and reputation, and not by title after specific training. The implementation of the model of training specialists through medical residency raised relevant questions: who would be the first professors/preceptors? And what to do with the professionals with notorious knowledge who already practiced as specialists?

From this perspective, the specialist title test can be considered a strategy that stems from our understanding of the technical and scientific training of physicians, in addition to being related to these historical dilemmas. However, the contemporary context presents new challenges. The increase in the number of graduates from medical schools was not associated with adequate planning on the needs of specialties and specialists. This occurs in a context of stagnation and decrease in the supply of medical residency programs in areas essential to the functioning of the Public Health System (SUS), combined with the decrease in the interest and demand of physicians for these programs and the ever-present possibility of practicing medicine without the continuity of training after graduation. 

The scenario is even more complex due to the rapid and accelerated production of scientific knowledge in the health area, the reorganization of areas of activity and fields of knowledge, the transformations in the world of work, and the intense international mobility of health professionals.

In Brazil, we have observed the emergence of a phenomenon of unregulated expansion of lato sensu specialization courses, or other specialized professional improvement courses (lasting less than 360 hours). Supported by contemporary pedagogical concepts, such as curricular flexibility, student-centricity, university autonomy, education through work, and training by skills, these courses adapt more quickly to the needs of the market and the health system. 

This adaptation is due to a much more flexible regulatory framework that requires educational institutions only to register the courses offered for lato sensu specializations. With the consolidation and democratization of distance learning tools, especially after the COVID-19 pandemic, the offers of informal courses available on virtual platforms or social networks have multiplied.

In a partial report of Medical Demography 2025, Scheffer et al. analyzed the specializations offered to physicians and registered with the Ministry of Education. In all, they evaluated 8,543 courses offered by 660 institutions, of which 2,148 (25%) are aimed only at doctors. The most offered specialties are endocrinology, dermatology, and psychiatry. Notably, 52.3% of the courses are offered online or mixed modality, with an average workload of 507.63 hours and an average cost of R$ 15,782.36^4^. With an average of 31 vacancies per course, we can estimate that 66,588 physicians are attending these specializations, a number that exceeds the 50,259 physicians in residency courses with occupied vacancies (Source: SisCNRM, Aug/24).

Although the search of Brazilian physicians for ways to specialize, qualify, and update is good news, the migration of the specialization format from residency to lato sensu specializations is a cause for concern. Medical demographics reveal a decrease in the demand for physicians through residency programs, a phenomenon that has not yet been fully clarified. The number of first-year residents in direct access programs fell from 15,499 in 2018 to 13,300 in 20225.

While numerous courses are designed with a competency-oriented approach, the existing regulatory framework restricted to the educational system does not ensure reliable evaluation of these competencies. This situation may pose a risk to the requirements of the Brazilian health system, affecting both the official public sector and the private complementary subsystem.

It is essential to consider that, despite the solidity and stability of the National Commission for Medical Residency (CNRM), the medical residency system also lacks an effective evaluation model for programs or residents. Although the programs are evaluated by the CNRM for authorization to operate, this evaluation is periodically reviewed and can be revoked upon complaint, but there is no institutional evaluation system that allows the proposing institutions and society to know the quality of the programs or their graduates. In addition, there is no resident evaluation system that makes it possible to monitor the acquisition of skills and offer security to society regarding the proficiency of trained specialists.

The Brazilian regulatory framework for the training of medical specialists does not provide mechanisms for dimensioning social needs, nor for regulating the supply of vacancies for specialties or areas of activity. There are also no evaluation mechanisms for professional certification or recertification. The reference title, commonly called the gold standard, which is medical residency, does not provide for structured formative or summative evaluations, nor final evaluation for authorization of professional practice or periodic reevaluation. This model is called “time-based”, where it is enough for the professional to complete the internship time to obtain certification.

It is vital to emphasize that the training of specialists must follow a structured model with attentive regulation to certification standards6 and that promotes competency-based education (CBME)7,8. This approach will ensure the acquisition of practical skills and a system of continuous assessment that ensures the proficiency of doctors in training. Thus, the urgency to review the regulatory framework that governs the training of specialists in Brazil becomes evident. The clear definition of competencies, aligned with the needs of SUS, can serve as a guide for professional regulation, evaluation, and certification.

In view of the current context and the transformations that are announced with the incorporation of artificial intelligence in health work, it is urgent to revisit the regulatory framework that governs training and professional practice in Brazil. This implies discussing and planning, based on needs and demands, access to and coverage of the specialist training system, its financing, the process of institutional and educational evaluation of the programs, and the continuous evaluation of residents, based on a model of dimensioning of social needs. 

For the construction of this regulatory framework, the concept of competencies that meet the needs of the population/SUS is the key that can guide its dimensioning, programmatic organization, and evaluation, as well as professional certification. The definition of competencies can also guide the regulation of specializations, offering parameters for authorization, proficiency assessment, and professional certification.
